# Clinical Scoring Systems in Predicting the Outcome of Acute Upper Gastrointestinal Bleeding; a Narrative Review 

**Published:** 2017-01-11

**Authors:** Hanieh Ebrahimi Bakhtavar, Hamid Reza Morteza Bagi, Farzad Rahmani, Kavous Shahsavari nia, Arezu Ettehadi

**Affiliations:** 1 Emergency Medicine Research Team, Tabriz University of Medical Sciences, Tabriz, Iran.; 2 Road Traffic Injury Research Center, Tabriz University of Medical Sciences, Tabriz, Iran.

**Keywords:** Hemorrhage, upper gastrointestinal tract, prognosis, mortality, decision support techniques

## Abstract

Prediction of the outcome and severity of acute upper gastrointestinal bleeding (UGIB) has significant importance in patient care, disposition, and determining the need for emergent endoscopy. Recent international recommendations endorse using scoring systems for management of non-variceal UGIB patients. To date, different scoring systems have been developed for predicting the risk of 30-day mortality and re-bleeding. We have discussed the screening performance characteristics of Baylor bleeding score, the Rockall risk scoring score, Cedars-Sinai Medical Center predictive index, Glasgow Blatchford score, T-score, and AIMS65 systems, in the present review.

Based on the results of this survey, there are only 3 clinical decision rules that can predict the outcome of UGIB patients, independent from endoscopy. Among these, only Glasgow Blatchford score was highly sensitive for predicting the risk of 30-day mortality and re-bleeding, simultaneously.

## Introduction

Prediction of the outcome and severity of upper gastrointestinal bleeding (UGIB), with 10% mortality rate, has significant importance in patient care, disposition, and determining the need for emergent endoscopy (1, 2). 

Some clinical criteria such as hemodynamic and mental status, volume of blood lost, and coagulopathy profile were used in determining the patient’s outcome (3). In addition, early upper gastrointestinal endoscopy, within the first 24 hours of emergency department (ED) arrival, is recommended for treatment as well as outcome prediction of these patients (1). Clots adhering to the ulcer, visible bleeding, and visible vessel in the ulcer are associated with high probability of re-bleeding. On the other hand, brown or black pigments on the ulcer and clean based ulcer are associated with low probability of re-bleeding (3, 4). Endoscopic triage of these patients could reduce duration of hospital stay and costs, but due to lack of endoscopy devices and absence of interventionists, it is not obtainable in the majority of EDs (5).

To date, different clinical scoring systems have been developed to predict the outcome and guide the management and disposition of these patients (3, 6). Recent international recommendations regarding management of non-variceal UGIB patients endorse using these systems for risk stratification in the first visit (7). 

Hereby, 6 of these scoring systems and their screening performance characteristics will be discussed comprehensively.

1. Endoscopy dependent scoring systems 

1 – 1. Baylor bleeding score (BBS) 

This scoring system was introduced by Saeed ZA et al. in 1993. They designed it for predicting the risk of re-bleeding in patients with UGIB (table 3). This scoring system contains three parts, namely pre-endoscopy, during endoscopy, and post-endoscopy. The score of ≥ 6 in the pre-endoscopy part and ≥ 11 in the total score have 100% sensitivity for predicting the risk of re-bleeding (10, 11). In another research in 1995, the same authors indicated that the score of ≤ 5 before endoscopy and ≤ 10 after endoscopy are associated with low re-bleeding risk. They finally revealed that BBS determines high-risk patients prone to re-bleeding after successful hemostatic endoscopy with high accuracy (12).

1 – 2. Rockall risk scoring score (RS) 

Rockall et al. introduced RS system in 1997. This score consists of two parts: pre-endoscopy, also known as clinical Rockall score, and post-endoscopy, which is called the Rockall risk score (table 4). They figured out that as the score increases, the chance of mortality or re-bleeding increases (2, 13). Different studies indicated that this system is practical, accurate, and a quick tool in prediction of re-bleeding and mortality risk (14-16). Evaluating the system in patients with non-variceal UGIB, Rahman MW showed that the score of ≤ 3 predicts low risk, while ≥ 8 is a predictor of high mortality risk. They also revealed the good performance index of RS system in UGIB patients’ triage (17). 

1 - 3. Cedars-Sinai Medical Center Predictive Index (CSMCPI) 

This scoring system has been introduced according to the suggestions by American Society of Endoscopy of the Digestive System in 1981 (18). It consists of four sections (table 5). The first one was related to endoscopic findings, the second dealt with the time interval between symptom initiation and hospitalization, the third section was related to the hemodynamic status of the patient, and finally the fourth section was related to the number of comorbidities. The total score of the patient was the sum of scores obtained from these four sections (18, 19). Patients with a score < 3 can be discharged from ED and others need hospitalization. These patients are re-evaluated after 24-72 hours based on endoscopy results. A validation study in 1997 showed that application of this scoring system with great accuracy, can lead to decreased duration of hospital stay (20). 

2. Clinical scoring systems (independent from endoscopy)

2 – 1a. Glasgow Blatchford score (GBS) 

Blatchford et al. studied the mortality rate of 1882 UGIB patients previously undergone endoscopy to introduce a new scoring system for predicting UGIB patients’ need for therapeutic interventions and blood transfusion as well as mortality risk, re-bleeding, and dropped hemoglobin levels after hospitalization (table 6) (21). A study evaluating the validity of this score in 2000 showed that patients with a score of zero belong to the low-risk group, and can be discharged from ED (22). Stanley et al. compared GBS and RS in a multicenter study and concluded that none of the patients categorized as low risk in the GBS scoring system, needed intervention during hospitalization and no mortality was observed in this group. Eventually, the authors concluded that application of GBS can contribute to reducing hospitalization rate and costs in low-risk patients (23). 

2 – 1b. Modified Glasgow Blatchford score (mGBS)

D.W. Cheng et al introduced a modified type of GBS system (mGBS). In this system, three variables related to syncope, melena, and comorbidities were omitted from the GBS, and the new scoring system was introduced based on the status of clinical and laboratory findings and patient symptoms. They concluded that the mGBS score could predict the outcome of patients with high accuracy. It is also more convenient to use compared to GBS (2).

2 – 2. T-score system

T-score system was designed based on the clinical status of UGIB patients before performing endoscopy (table 7). Patient’s general appearance, number of comorbid diseases, pulse rate, systolic blood pressure, and hemoglobin level are among T score variables. A score < 6 indicates high-risk status (T1), a score between 7 and 9 shows moderate-risk status (T2), and a score of ≥ 10 reveals low-risk status (T3). Good clinical conditions include a patient without weakness or orthostatic hypotension who have ≤ 1 comorbidity. Validation studies concluded that this score could determine the need of UGIB patients for early endoscopy with an accuracy equal to GBS (9, 24).

2 – 3. AIMS65 system

Recently, a simple score was introduced by Saltzman JR et al. for evaluating the prognosis of UGIB patients. It includes five variables: age over 65, systolic blood pressure lower than 90 mmHg, altered level of consciousness, international normalized ratio (INR) higher than 1.5, and serum albumin lower than 3 g/dL. The patient would receive one score for presence of each variable. Eventually, mortality rate was estimated to be 0.3% for score 0, 1.2% for score 1, 5.3% for score 2, 10.3% for score 3, 16.5% for score 4, and 24.5% for score 5. Scores of 0-1 and 2-5 are related to low-risk and high-risk patients, respectively. Saltzman JR et al. concluded that this score has high accuracy in prediction of in-hospital mortality, length of hospital stay, and reduction of the hospitalization cost in patients with UGIB (5). Hyett BH et al. compared AIMS65 and GBS and concluded that AIMS65 is more accurate in prediction of mortality in comparison with GBS, while GBS is more accurate in estimation of need for blood transfusion. Both scores were similar in prediction of other outcomes (25). Yaka E et al. also compared AIMS65 and GBS, and concluded that GBS has a lower sensitivity in predicting the need for emergent intervention in comparison with AIMS65 (5).

## Discussion

In this narrative review, we evaluated 6 different scoring systems to predict the outcome of patients with acute UGIB. Each of these systems used different variables in predicting the outcome of acute UGIB patients. 

Based on the findings of this review, only AIMS65, GBS, and T score were designed to determine the outcome of UGIB patients without needing emergency endoscopy. Furthermore, only AIMS65 score determines the outcome of patients disregarding comorbidities and only based on the patient’s current clinical status.

Some researchers want to predict the patients’ outcome without considering the comorbidities or even endoscopy results, so they have modified some scoring system such as GBS or Clinical Rockall Score.

A weak point of CSMCPI score was that definition of hemodynamic status was vague in this system (8). 

Table 1 and 2 summarize the screening performance characteristics of these scoring systems in the risk prediction of re-bleeding and 30-day mortality. In the case of predicted probability of re-bleeding, RS, BBS, and CSMCPI have similar values and these systems do not have any significant differences in prediction of re-bleeding, but AIMS65 score has a higher specificity and positive predictive value (82%, and 89% respectively), in this regard. These characteristics represent the great value of this system compared to others. In addition, AIMS65 and T score have the same value in predicting 30-day mortality.

Another difference of AIMS65 with other systems is that, unlike the others, it only assesses the current clinical status of the patient, regardless of underlying diseases.

Despite the presence of all these scoring systems, none is routinely used in emergency departments. This might be due to various reasons such as lack of validation studies, not being user-friendly, and not believing in evidence-based medicine. It seems that we need further validation studies prior to implementing these clinical decision rules in our routine practice.

**Table 1:** Screening performance characteristics of scoring systems in predicting re-bleeding risk in upper gastrointestinal bleeding

**Scores**	**Sensitivity**	**Specificity**	**PPV**	**NPV**
**GBS**	1.00	0.12	0.07	1.00
**BBS**	1.00	0.26	0.10	1.00
**CSMCPI**	0.88	0.32	0.10	0.97
**RS**	1.00	0.28	0.10	0.99
**AIMS65**	0.35	0.82	0.89	0.23

PPV: Positive predictive value, NPV: Negative predictive value, GBS: Glasgow Blatchford score, BBS: Baylor bleeding score, CSMCPI: Cedars-Sinai Medical Center predictive index, RS: Rockall risk scoring score.

**Table 2:** Screening performance characteristics of scoring systems in predicting 30-day mortality in upper gastrointestinal bleeding

**Scores**	**Sensitivity**	**Specificity**	**PPV**	**NPV**
**GBS**	0.97	0.13	0.14	0.97
**BBS**	0.97	0.27	0.14	0.98
**CSMCPI**	0.97	0.29	0.15	0.99
**RS**	0.96	0.27	0.14	0.98
**T score**	0.71	0.80	0.78	0.73
**AIMS65**	0.35	0.82	0.89	0.23

PPV: Positive predictive value, NPV: Negative predictive value, GBS: Glasgow Blatchford score, BBS: Baylor bleeding score, CSMCPI: Cedars-Sinai Medical Center predictive index, RS: Rockall risk scoring score.

**Table 3:** Baylor Bleeding Score and interpretation

**Score**	**Pre-endoscopy Score (range: 0 - 15)**			**Endoscopy Score (range: 0 – 9)**	
	**Age (years)**	**No. of illnesses**	**Severity of illnesses**	**Site of bleeding**	**Stigmata of bleeding**
**0**	< 30	0			
**1**	30 - 49	1 or 2			Clot
**2**	50 - 59				
**3**	60 - 69				Visible vessel
**4**		3 or 4	Chronic	Posterior bulb	
**5**	≥ 70	≥ 5	Acute		Active bleeding

Total score is the sum of scores obtained for each item, which ranges from 0 to 24. Score ≥ 6 in pre-endoscopic phase and total score ≥ 11 indicate high-risk patients for re-bleeding.

Example: A 34-year-old patient (score: 1) with chronic (score: 4) hepatic failure (score: 1) and adhering clot (score: 1) on ulcer in endoscopy has a score of 7 (1 + 4 + 1+ 1).

**Table 4:** Rockall risk scoring score and interpretation

**Variable**	**Score**			
	**0**	**1**	**2**	**3**
**Age (year)**	< 60	60 - 79	≥ 80	
**Shock stage**				
SBP (mmHg)	≥ 100	≥ 100	< 100	
PR (1/minute)	< 100	≥ 100	-	
**Comorbidity**	No major comorbidity		Cardiac failure, ischemic heart disease, any major comorbidity	Renal failure, liver failure, disseminated malignancy
**Diagnosis**	Mallory-Weiss tear, no lesion identified and no SRH	All other diagnosis	Malignancy of upper GI tract	
**Major SRH**	None or dark spot only		Blood in upper GI tract, adherent clot, visible or spurting vessel	

SBP: Systolic blood pressure, PR: Pulse rate, GI: Gastrointestinal, SRH: Signs of recent hemorrhage.

Range of score is 0-11. Score of ≤ 3 predicts low mortality risk, while ≥ 8 is a predictor of high mortality risk.

**Table 5:** Cedars-Sinai Medical Center predictive index and interpretation

**Score**	**Endoscopic findings**	**Time** *	**Hemodynamics**	**Comorbidities**
**0**	PUD (no SRH), Mallory-Weiss tear (NB), Erosive disease (no SRH), Normal finding	> 48	Stable	≤ 1
**1**	PUD (spot/clot), Erosive disease (SRH), Angiodysplasia	< 48	Intermediate	2
**2**	PUD (VVNB/SRH)	In hospital	Unstable	3
**3**				≥ 4
**4**	Persistent UGIH, Varices, UGI cancer			

Score range is 0 to 11. Patients with a score < 3 can be discharged from ED and others need hospitalization. The criteria of hemodynamic classification was not clearly defined.

* Time means the interval between the initiation of bleeding and arrival to emergency department (hour), NB: Non-bleeding, PUD: Peptic ulcer disease, SRH: Signs of recent hemorrhage, UGI: Upper gastrointestinal, UGIH: Upper gastrointestinal hemorrhage, VVNB: Visible vessel, non-bleeding.

**Table 6:** Glasgow Blatchford score

**Admission risk markers**	**Score **
**Blood urea nitrogen level (mg/dl)**	
≥ 18.2 to < 22.4	2
≥ 22.4 to < 28	3
≥ 28 to < 70	4
≥ 70	6
**Hemoglobin level for men (g/dl)**	
≥ 12 to < 13	1
≥ 10 to < 12	3
< 10	6
**Hemoglobin level for women (g/dl)**	
≥ 10 to < 12	1
< 10	6
**Systolic blood pressure (mmHg)**	
≥ 100 to < 109	1
≥ 90 to < 99	2
< 90	3
**Other markers**	
Pulse rate ≥ 100 beats/min	1
Presentation with melena	1
Presentation with syncope	2
Hepatic disease*	2
Heart failure#	2

Range of score is 0-29. Score ≥ 0 is high-risk group.

* Known history, or clinical and laboratory findings of chronic or acute hepatic disease,

# Known history, or clinical and echocardiographic findings of heart failure.

**Table 7:** T-score

**Clinical parameter**	**Score**		
	**1**	**2**	**3**
**General conditions**	Poor	Intermediate	Good
**Pulse (beats/minute)**	> 110	90 - 110	< 90
**Systolic blood pressure (mmHg)**	< 90	90 - 110	> 110
**Hemoglobin levels (g/dl)**	≤ 8	9 - 10	> 10

General condition was defined based on the patient’s number of comorbidities.

Poor condition was associated with ≤ 3 comorbidities or impending to shock.

Good condition means a patient is without weakness or orthostatic hypotension and has ≤ 1 comorbidity. Intermediate condition includes patients with conditions between the mentioned two groups.

A score < 6 indicates high-risk (T1), a score between 7 and 9 shows moderate-risk (T2), and a score of ≥ 10 reveals low-risk patients (T3) for detection of major findings in endoscopy.

## Conclusion

Based on the results of this survey, there are only 3 clinical decision rules that can predict the outcome of UGIB patients, independent from endoscopy. Among these, only Glasgow Blatchford score was highly sensitive for predicting the risk of 30-day mortality and re-bleeding simultaneously. 
